# Impact of Untreated Obstructive Sleep Apnea on Left and Right Ventricular Myocardial Function and Effects of CPAP Therapy

**DOI:** 10.1371/journal.pone.0076352

**Published:** 2013-10-11

**Authors:** Christoph Hammerstingl, Robert Schueler, Martin Wiesen, Diana Momcilovic, Stefan Pabst, Georg Nickenig, Dirk Skowasch

**Affiliations:** University of Bonn, Department of Internal Medicine II, Cardiology, Pulmonology and Angiology, Bonn, Germany; University of Buenos Aires, Faculty of Medicine. Cardiovascular Pathophysiology Institute, Argentina

## Abstract

**Background:**

Obstructive sleep apnea (OSA) has deteriorating effect on LV function, whereas its impact on RV function is controversial. We aimed to determine the effect of OSA and continuous positive airway pressure (CPAP) treatment on left and right ventricular (LV, RV) function using transthoracic echocardiography (TTE) and 2 dimensional speckle tracking (2D ST) analysis of RV deformation capability.

**Methods and Results:**

82 patients with OSA and need for CPAP therapy were prospectively enrolled and underwent TTE at study inclusion and after 6 months of follow up (FU). Multivariate regression analysis revealed an independent association between baseline apical right ventricular longitudinal strain (RV-Sl), BMI and the severity of OSA (apical RV-Sl: P = 0.0002, BMI: P = 0.02). After CPAP therapy, LV functional parameters (LVEF: P<0.0001, LV performance index: P = 0.03, stroke volume: P = 0.042), and apical RV-Sl (P = 0.001) improved significantly. The effect of CPAP therapy was related to severity of OSA (LVEF: AHI 5–14, 66.4±8.8%, 68.5±10.6% [P = ns]; AHI 15–30∶59.8±7.7%, 68.6±9.3% [P = 0.002]; AHI>30∶54.1±12.4%, 68.2±13.6%[P<0.0001]; apical RV-Sl: AHI 5–14: −17.3±8.7%, −16.0±10.8% [P = ns], AHI 15–30: −9.8±6.0%, −15.4±10.9% [P = 0.028], AHI>30: −6.3±5.7%, −17.9±11.2% [P<0.0001]).

**Conclusions:**

OSA seems to have deteriorating effect on LV and RV function. We found a beneficial effect of CPAP on LV and RV functional parameters predominately in patients with severe OSA. 2D speckle tracking might be of value to determine early changes in global and regional right ventricular function.

## Introduction

Obstructive sleep apnea (OSA) is a frequent sleep-related breathing disorder with an incidence of 5–20% in the middle-aged population in Europe and Northern America [1], [2]. Pathophysiologic consequences of OSA are increased sympathetic activity, hypoxia, hypercapnia, increased left ventricular afterload and acute arterial hypertension [3]. Recent studies have shown that OSA is an independent risk factor for cardiovascular mortality and morbidity [4]. In clinical practice, it might be difficult to evaluate the effects of OSA on myocardial function because many of the risk factors for OSA, such as obesity, male gender, and age may contribute to both, OSA and cardiovascular disease [5].

Using conventional Doppler echocardiography, several studies have recently reported the detrimental effect of untreated severe OSA on systolic and diastolic left ventricular (LV) function [6], [7]. The effect of OSA on right ventricular (RV) function and its reversibility under effective therapy is not well investigated. Furthermore, the usefulness of two dimensional speckle tracking (2D ST), a novel ultrasound based technique for the determination of regional and global myocardial deformation properties [8], to visualize OSA related changes in RV function is unclear.

The aims of this prospective cohort study were (i) to investigate the impact of OSA and its severity on left and right ventricular function measured with echocardiography and two-dimensional strain analysis and (ii) to determine the effect of effective OSA therapy on measurable left/right ventricular functional parameters.

## Methods

### Patients and Follow Up

Patients admitted between May 2009 and December 2009 to the Department of Pneumology of our hospital, for OSA screening and initiation of continuous positive airway pressure therapy (CPAP) were included in the study. Clinical follow-up examinations were scheduled after 1 and 6 months for the adjustment of CPAP therapy. Echocardiography for the detection of CPAP related changes in LV/RV function was planned at study initiation and after 6 months (±14 days) of follow up.

According to current research we hypothesized that OSA has a deteriorating effect on LV function (defined as significant decrease in LV EF) and that after a 6 months CPAP therapy the impairment could in part be ameliorated. Furthermore we assumed RV function to be likewise impaired by OSA (defined as significant decrease in RV strain) and likewise ameliorated by CPAP. *Study endpoints* were (i) prevalence of echocardiographically detectable pathologic myocardial left and right ventricular functional parameters in patients undergoing OSA screening before initiation of CPAP therapy, and (ii) the evaluation of changes in measurable LV/RV functional parameters after OSA therapy with CPAP.

All patients had to provide written informed consent prior to study inclusion; the study was approved by local ethics committee and was in accordance with the Declaration of Helsinki. Exclusion criteria were presence of predominant central sleep apnea (CSA), non compliance to CPAP (<4 h use/night) and AHI<5. However, the number of patients not compliant with CPAP or AHI<5 and willing to comply with the study protocol was too small to serve as a meaningful control group.

### OSA Diagnosis and Initiation of CPAP Therapy

All patients underwent an overnight polysomnographic study at baseline (SOMNOlab, Weinmann, Hamburg) and polygraphy controls after one week and 6 months (Embletta, Medcare Flaga Hf, Reykjavik).

On the day of the overnight sleep study, classic baseline characteristics were assessed (***Table 1***). Epworth sleepiness scale (ESS) was obtained to assess daytime sleepiness. Thoracoabdominal movements were measured by calibrated respiratory inductance plethysmograph and oxyhemoglobin saturation was obtained by fingertip oximetry. The mean lowest oxyhemoglobin saturation (SaO2) during sleep was calculated by averaging the lowest SaO2 for each 30-s episode during sleep. OSA was defined as an absence of airflow for at least 10 s and hypopneas as a >50% reduction in airflow from the baseline level for at least 10 s with 3% decrease in oxygen saturation, during which there were paradoxical thoracoabdominal movements. The diagnosis of OSA at baseline was made by standard attended polysomnography (SOMNOlab, Weinmann, Hamburg) device: The electroencephalogram, electrooculogram, and electromyogram of chin muscles, as well as ECG were simultaneously recorded; oral-nasal airflow (with thermal and pressure sensing device), thoraco-abdominal respiratory movements, body position, snoring, and oximetry were also obtained. Then, the patients underwent a titration of the allocated CPAP device during a second overnight, in–laboratory, attended polysomnography. Airway pressure was manually modulated from 4 cm H_2_O to the effective pressure, with a maximum of 12 cm H_2_O. The appropriate fixed pressure was chosen as the pressure abolishing or significantly decreasing obstructive events. Patients underwent polygraphy controls after one week and 6 months (Embletta, Medcare Flaga Hf, Reykjavik). According to current guidelines, subjects with more than five obstructive apneas and hypopneas were considered as suffering from OSA. In addition, central apneas and snoring were measured. Analysis and interpretation of sleep study data were performed without knowing the patients clinical condition.

**Table 1:** Demographic and echocardiographic characteristics of the study group.

	All patients atbaseline (mean ±SD) (n = 82)	All patients at 6months FU (mean ±SD) (n = 82)	Group 1 AHI5–14 (n = 29)	Group 2 AHI15–30 (n = 24)	Group 3 AHI>30(n = 29)	*p-value*
Age [years]	63.3±11.5		61.8±13.0	66.3±10.5	62.5±10.7	ns
Male gender	52 (63.4%)	52 (63.4%)	21 (72.4%)	13 (54.1%)	17 (58.6%)	ns
BMI [kg/m^2^]	30.7±5.5	30.7±6.4	28.9±4.9	30.4±4.5	32.9±6.3	0.02
AHI [n/h]	31.4±26.8	5.6±7.1†	9.0±2.8	22.0±4.4	61.7±22.7	<0.0001
ODI	22.7±23.0	7.3±10.1†	8.4±8.7	19.2±10.2	40.5±28.7	<0.0001
ESS	10.0±5.4	7.5±4.5†	8.5±4.1	9.4±4.9	13.6±4.9	0.03
Hypertension	44 (53.6%)	44 (53.6%)	13 (44.8%)	15 (62.5%)	16 (55.1%)	ns
CHF	16 (19.5%)	16 (19.5%)	4 (13.8%)	4 (16.6%)	7 (24.1%)	ns
Diabetes mellitus	10 (12.2%)	10 (12.2%)	1 (3.4%)	4 (16.6%)	5 (17.2%)	ns
History of stroke	4 (4.9%)	4 (4.9%)	2 (6.8%)	2 (8.3%)	0 (0%)	ns
CAD	11 (13.4%)	11 (13.4%)	3 (10.0%)	5 (20.8%)	3 (10.3%)	ns
Smoking	30 (36.6%)	30 (36.6%)	10 (34.5%)	7 (29.1%)	12 (41.3%)	ns
HLP	30 (36.6%)	30 (36.6%)	9 (31.0%)	12 (50%)	9 (31%)	ns
***Medication***						
Aspirin	25 (30.5%)	25 (30.5%)	7 (24.1%)	9 (37.5%)	9 (31%)	ns
Beta blocker	25 (30.5%)	25 (30.5%)	8 (27.6%)	7 (29.2%)	10 (34.5%)	ns
ACEI/ARB	25 (30.5%)	25 (30.5%)	6 (20.7%)	9 (37.5%)	10 (34.5%)	ns
Diuretics	26 (31.7%)	26 (31.7%)	8 (27.6%)	10 (41.7%)	8 (27.6%)	ns
Statin	18 (22%)	18 (22%)	6 (20.7%)	7 (29.2%)	5 (17.2%)	ns
Oral anticoagulant	5 (6.1%)	5 (6.1%)	1 (3.4%)	3 (12.5%)	1 (3.4%)	ns
***Echocardiography***						
LVEF [%]	60.7±8.4	63.2±7.2†	65.0±6.9	59.5±6.9	57.5±5.6	<0.0001
sPAP [mmHg]	16.9±11.2	16.7±11.6	18.6±12.3	15.7±10.1	16.4±11.0	ns
IVSd [cm]	1.3±0.2	1.3±0.4	1.3±0.3	1.3±0.2	1.3±0.3	ns
SV [ml]	66.0±23.9	67.7±21.9	66.0±23.9	68.7±23.7	62.6±21.5	ns
MV e′/a′	0.8±0.9	0.8±0.4	0.8±0.9	0.9±0.3	0.9±0.4	ns
LV MPI	0.4±0.2	0.4±0.2	0.4±0.2	0.4±0.2	0.5±0.1	ns
e/e′	11.0±6.2	10.1±3.6	9.9±2.7	10.3±3.2	12.7±9.5	ns
RV MPI	0.4±0.3	0.4±0.2	0.3±0.2	0.4±0.4	0.3±0.3	ns
TDI TKS [m/s]	0.1±0.02	0.4±1.9	0.1±0.02	0.1±0.02	0.1±0.03	ns
TAPSE [mm]	24.8±5.9	24.3±5.9	24.2±6.5	24.9±6.0	25.1±6.4	ns
2D global RV-Sl [%]	−16.9±7.5	−17.6±8.6	−21.5±6.3	−14.3±5.3	−14.5±8.2	<0.0001
2D apical RV-Sl [%]	−11.3±8.4	−15.0±6.8†	−17.3±8.7	−9.8±6.0	−6.3±5.7	<0.0001
2D medial RV-Sl [%]	−16.9±8.2	−15.6±8.0	−19.8±5.6	−14.9±6.8	−15.7±10.7	ns
2D basal RV-Sl [%]	−22.7±13.3	−21.5±12.8	−27.4±13.6	−18.2±8.7	−21.6±14.9	0.03

† significance of changes when comparing baseline and follow up measurements <0.05; 2D RV-Sl, two dimensional right ventricular longitudinal strain; ACEI, angiotensin converting enzyme inhibitors; AHI, apnea hypopnea index; ARBI, angiotensin receptor blocker; CAD, coronary artery disease; CHF, chronic heart failure; BMI, body mass index; e/e′, early/early′; ESS, Epworth sleepiness scale; HLP, hyperlipoproteinemia; IVSd, diastolic interventricular septum thickness; LVEF, left ventricular ejection fraction; LV/RV MPI, left ventricular/right ventricular myocardial performance index; MV e′/a, mitral velocity early′/atrial; ns, not statistically significant; ODI, oxygen desaturation index; SD, standard deviation; sPAP, systolic pulmonary artery pressure; SV, stroke volume; TAPSE, tricuspid annular plane systolic excursion; TDI TKS, tricuspid annular systolic tissue Doppler velocity.

CPAP was recommended to all patients who had more than 30 episodes of apnea or hypopnea per hour of sleep. CPAP was also recommended if the AHI was between 5 and 30 and the patient complained of severe daytime sleepiness that interfered with daily activities.

Compliance with CPAP was checked by readings of the built in time counter of the patient’s device. Only those patients for whom calculation of the time of use per night gave a value higher than four hours were considered compliant with. The adherence to CPAP in the whole patient cohort was 4.8±1.4 hours per night, whereas the time of used CPAP showed to be 6.5±1.1 hours per night in the study cohort. Effective OSA therapy was defined as ≥25% drop in AHI or AHI<5 after 6 months of CPAP treatment and mean reduction in Epworth Sleepiness Score of 3 points [9], [10].

### Transthoracic Echocardiography

Each patient had a complete standardized two-dimensional transthoracic echocardiography study for the determination of LV/RV functional parameters and dimensions according to the recommendations of the American Society of Echocardiography [11], [12] using a commercially available ultrasound scanner (Vivid 7, General Electric Medical Health, Waukesha, Wisconsin, USA; iE 33, Philips Medical Systems, Koninklijke N.V.) with a 2.5-MHz phased-array transducer. Echocardiographic views, including apical four- and two-chamber views (4CV, 2CV), with the patient in the left lateral decubitus position, were obtained in two-dimensional and colour tissue Doppler imaging (TDI) modes. Mitral inflow velocities were recorded by standard pulsed-wave Doppler at the tips of the mitral valve leaflets in an apical 4CV. TDI derived systolic and diastolic velocities were obtained from the septal mitral and lateral tricuspidal valve annuli.

LV and RV myocardial performance indices (MPI) were assessed, following the original approach of Tei and colleagues and were therefore calculated as the ratio between the sum of times of the isovolumetric periods and ejection time for the right ventricle [13], [14]. Echo systolic pulmonary artery pressure (sPAP) was estimated by measuring the peak systolic tricuspid regurgitant velocity flow from continuous wave Doppler if applicable. Values of sPAP are provided in mmHg, without addition of central venous pressure.

For the determination of longitudinal RV contractility, tricuspidal annular systolic excursion (TAPSE) was measured in the four-chamber view [15] as well as systolic tricuspid annulus velocities with TDI. All echocardiographic studies were performed by one single experienced cardiologist. Interpretation of the echo data was performed by echo experienced cardiologists, who were blinded to the clinical information when analysing the data.

### 2D-speckle-tracking Analysis of Right Ventricular Deformation Capabilities

Two cine loops from apical four chamber view were digitized and stored in an echocardiographic imaging server (XCELERA, Philips Medical Systems, Koninklijke N.V.). Offline 2D-ST-analyses of the gray scale images obtained by 2D echocardiography were done using commercially available software (TomTec Imaging Systems GmbH, Unterschleissheim, Germany). The endocardium of the free RV wall was manually traced starting from the lateral tricuspdial annulus to RV apex, and was tracked by the 2D strain software along the border throughout two cardiac cycles. Accuracy of border tracking was manually verified and adjusted if needed. The free right ventricular wall was segmented visually in a basal, midventricular and apical segment. For the determination of OSA related changes in RV contractility, we compared global as well as regional RV functional parameters of the RV free wall.

### Statistical Analysis

Exploratory data analysis was performed and no adjustment was made for multiple tests. Two-tailed P-values were calculated and considered to be significant if ranging below 0.05. Continuous data were expressed as mean values ± standard deviation. For comparisons between groups normalcy tests were done for each parameter. Continuous variables were tested for differences with the Kruskal-Wallis or One-way ANOVA test, when comparing more than two groups. For categorical variables, the Chi square or Fisher’s exact test were used for further analysis. Demographic and echocardiographic variables were evaluated for their association with severity of OSA; a variable was selected for further multivariate analysis if the level of significance in univariate logistic regression was ≤0.2 and tested in a stepwise regression model, using the severity of OSA as the dependent variable.

For the assessment of inter-observer agreement, two different investigators analyzed 10 randomly chosen patients independently. For the evaluation of inter- and intraobserver reproducibility of strain measurements intra-class correlation coefficients were calculated with good agreement defined as having a coefficient >0.8.

Statistics were performed using SPSS for Windows (PASW statistic., Version 17.0.2, SPSS Inc., Chicago, Illinois, USA) and MedCalc statistical software (MedCalc Software, Version 11.4.1.0, Mariakerke, Belgium).

## Results

### Clinical Characteristics of Patients Undergoing Polysomnography

150 patients, submitted to our clinic because of suspected OSA, were screened. Only in 90 of patients significant OSA requiring CPAP therapy could be found. 5 of those declined participation in the study. 3 patients did not attend the follow up examinations and were therefore excluded from the final analysis. 82 consecutive patients with polysomnographic proven OSA were included in the study.

**Figure 1:**
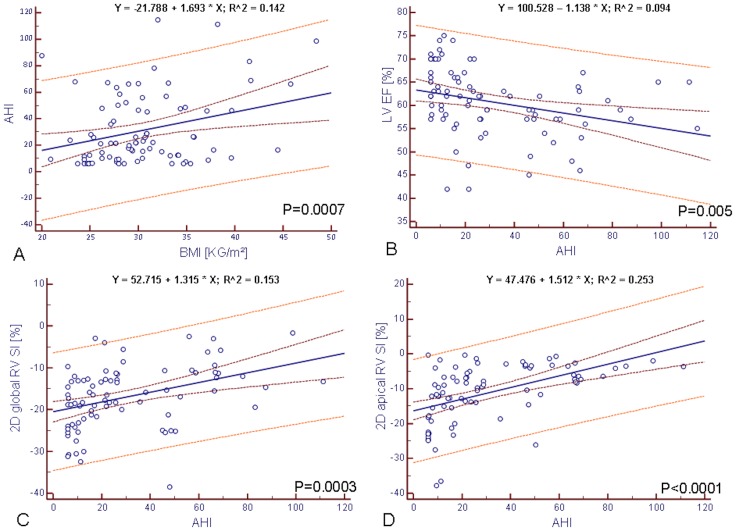
Scatter plot - Correlation of AHI to baseline data. (A) Correlation of AHI to BMI, (B) Correlation of AHI to LVEF, (C) Correlation of AHI global RV-Sl, (D) Correlation of AHI to apical RV-Sl. 2D RV-Sl, two dimensional right ventricular longitudinal strain; AHI, apnea hypopnea index; BMI, body mass index; LVEF, left ventricular ejection fraction.

Clinical and baseline characteristics of these patients are presented in ***Table 1*** **.** Overall, patients presented significant levels of daytime sleepiness (ESS 10.07±5.42) and severe OSA (AHI 30.75±5.5 n/h). Body mass index (BMI, 31.45±26.83 kg/m^2^) correlated significantly to the severity of the sleep disordered breathing (P = 0.0007) (***Figure 1a***).

Patients were divided into three groups according to the severity of OSA following the recommendation of the American Academy of Sleep Medicine guidelines [9], [16] (***group 1***: AHI 5–14, n = 29; ***group 2***: AHI 15–30, n = 24; ***group 3***: AHI>30, n = 29).

Patients in group 3 were more often obese, had significantly lower left ventricular ejection fraction and stroke volume. Values for global, apical and basal longitudinal strain were lower as compared to patients with moderate or low AHI score (***Table 1***).

### Comparison of Baseline Demographic and Echocardiographic Data According to the Severity of OSA

Severity of OSA at baseline was significantly correlated with a decrease in measurable systolic LV function (P = 0.005) (***Figure 1b***), whereas parameters for the determination of LV diastolic function were not significantly altered in patients with severe OSA.

Global and regional RV deformation properties derived from 2D RV longitudinal strain were significantly reduced in patients with higher AHI and correlated well to the severity of OSA (***Figure 1c,d*** ***,*** ***Table 1***). Conventional right functional parameters such as TAPSE, and right ventricular performance index (RV MPI) were not significantly decreased in patients with increasing AHI (***Table 1***).

Cardiovascular co morbidities or clinical risk factors, such as hypertension, coronary artery disease, or diabetes were not associated with the rate of apnea hypopnea episodes.

**Figure 2:**
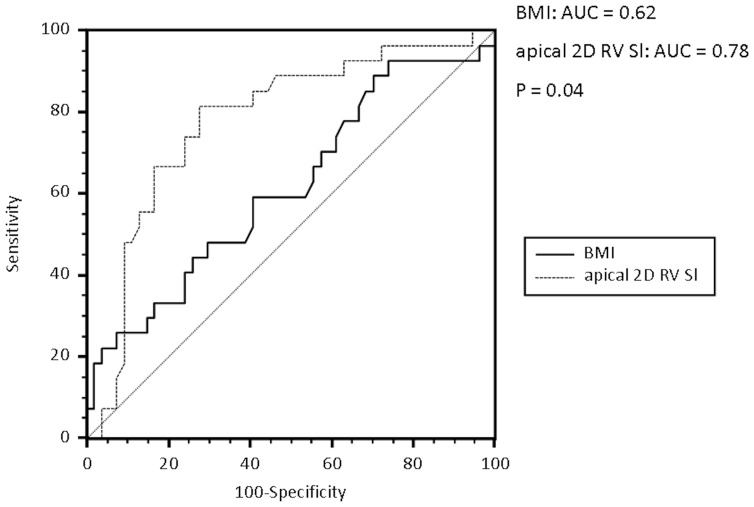
Comparative receiver operating characteristic (ROC) curves analysis of BMI and apical 2D RV-Sl for the identification of patients with an AHI>30. 2D RV-Sl, two dimensional right ventricular strain; AHI, apnea hypopnea index; P, significance of difference between the two curves.

After multivariate stepwise regression analysis of all echocardiographic parameters and baseline demographics (***Table S1***), only apical RV-Sl and BMI were independently associated with severe OSA (apical RV-Sl, P = 0.0002, BMI, p = 0.02). Severe OSA was defined as AHI>30, according to definitions by the American Academy of Sleep Medicine (9). These variables entered comparative receiver-operated-characteristic-curve (ROC) analysis (***Figure 2***) which identified apical RV-Sl (sensitivity: 81.5%, specificity: 72.2%, cut-off: −8.4) to have a stronger association with AHI>30 when compared to BMI (sensitivity: 46.4%, specificity: 74.1%, cut-off: 31.5).

Additionally, oxygen desaturation index (ODI) was significantly correlated with clinical and echocardiographic baseline data: r = 0.72, (P<0.0001) for correlation with AHI, r = 0.4 (P<0.0001) for global 2D RV-Sl, r = 0.36 (P = 0.001) for apical 2D RV-Sl, r = 0.35 (P = 0.001) for BMI, r = −0.17 (P = 0.13) for LV EF. These correlations were similar to correlations with AHI.

### Comparison of Echocardiographic Parameters in Patients before and after CPAP Therapy

The patients included in the analysis (age 63.35±11.54 years, 63.41% male) were all highly compliant to CPAP; it was used 6.5±1.1 hours per night (overall hours used 1988±1307.55/6 months).

AHI index and ESS decreased relevantly as evidence for clinical and subjective amelioration of obstructive sleep apnea syndrome (AHI: 31.45±26.83 vs. 5.63±7.0, P<0.0001; ESS 10.07±5.42 vs. 7.5±4.45, P<0.0001).

**Figure 3:**
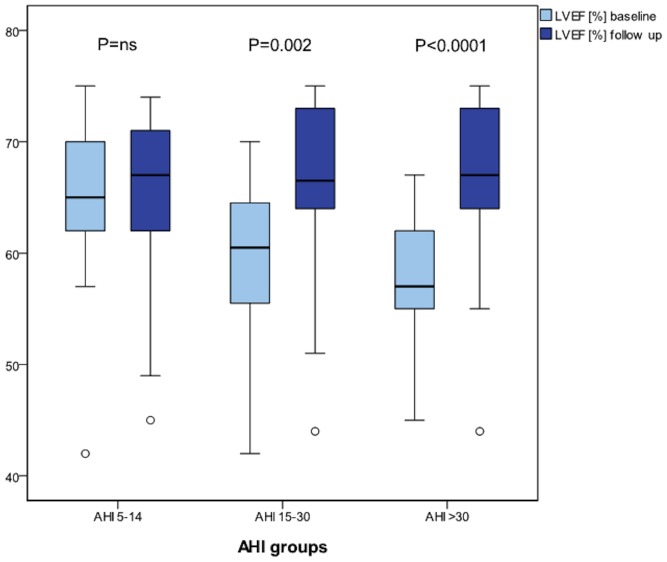
Boxplot - Development of left ventricular ejection fraction after CPAP according to AHI groups. AHI, apnea hypopnea index; LVEF, left ventricular ejection fraction; ns, not significant.

**Figure 4:**
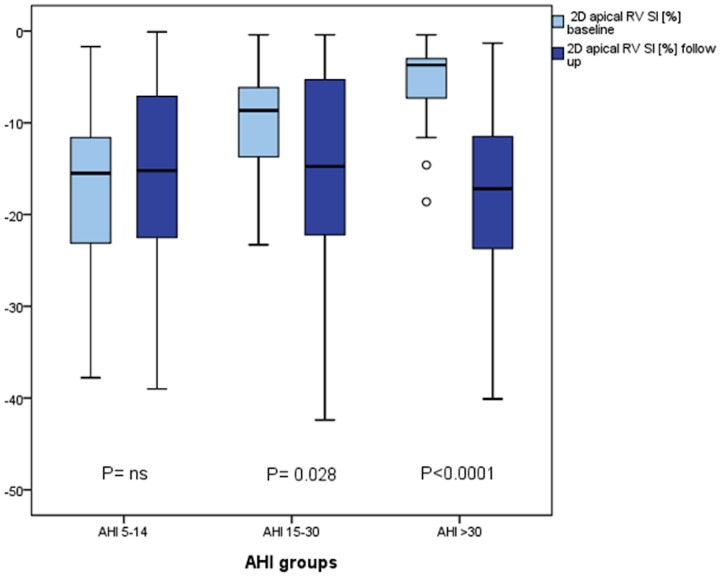
Boxplot - Development of apical RV-Sl after CPAP according to AHI groups. 2D RV-Sl, two dimensional right ventricular strain; AHI, apnea hypopnea index; ns, not significant.

After 6 months of treatment with CPAP therapy, we found a significant increase in systolic LVEF (***Figure 3***), LV MPI, LV stroke volume and apical RV-Sl (***Figure 4*** ***,*** ***Table 2***). Global right ventricular function parameters, TAPSE, e/e′ value, RV MPI and interventricular septum diameter, did not change relevantly over the treatment period (***Table 2***).

**Table 2:** Echocardiographic and clinical parameters before and after 6 months of CPAP treatment.

	Baseline(n = 82)	After CPAP (n = 82)	*p value*
AHI [n/h]	31.4±26.8	5.6±7.1	<0.0001
ESS	10.0±5.4	7.5±4.5	<0.0001
LVEF [%]	60.7±8.4	63.2±7.2	0.001
sPAP [mmHg]	16.9±11.2	16.7±11.6	ns
IVSd [cm]	1.3±0.2	1.3±0.4	ns
SV [ml]	66.0±23.9	67.7±21.9	ns
MV e′/a′	0.8±0.9	0.8±0.4	ns
LV MPI	0.4±0.2	0.4±0.2	ns
e/e′	11.0±6.2	10.1±3.6	ns
RV MPI	0.4±0.3	0.4±0.2	ns
TAPSE	24.8±5.9	24.3±5.9	ns
2D global RV-Sl [%]	−16.9±7.5	−17.6±8.5	ns
2D apical RV-Sl [%]	−11.3±8.4	−15.0±6.8	0.001
2D medial RV-Sl [%]	−16.9±8.2	−15.6±8.0	ns
2D basal RV-Sl [%]	−22.7±13.3	−21.5±12.8	ns

2D RV-Sl, two dimensional right ventricular longitudinal strain; AHI, apnea hypopnea index; CPAP, continuous positive airway pressure; e/e′, early/early′; ESS, Epworth sleepiness scale; IVSd, diastolic interventricular septum thickness; LVEF, left ventricular ejection fraction; LV/RV MPI, left ventricular/right ventricular myocardial performance index; MV e′/a, mitral velocity early′/atrial; ns, not statistically significant; SD, standard deviation; sPAP, systolic pulmonary artery pressure; SV, stroke volume; TAPSE, tricuspid annular plane systolic excursion; TDI TKS, tricuspid annular systolic tissue Doppler velocity.

Interestingly, in patients with severe OSA and AHI over 30, the benefit from CPAP therapy was pronounced when compared to patients with lower rates of AHI concerning RV and LV functional parameters. In those patients, even global RV-Sl improved after FU. (LV-EF: group 1: P = ns; group 2: P = 0.002; group 3: P<0.0001; global RV-Sl: group 1: P = ns; group 2: P = ns; group 3: P = 0.02; apical RV-Sl: group 1: P = ns; group 2: P = 0.028; group 3: P<0.0001).

### Feasibility and Reproducibility of Regional and Global RV-function Imaging

2D RV-Sl showed a good intra- and inter-observer agreement, which was more robust, when compared to 2D ST regional function analysis (***Table 3***).

**Table 3:** Intraobserver and interobserver reproducibility of apical and global 2D RV-Sl assed by 2 dimensional speckle tracking imaging.

	Intraobserver			Interobserver		
	Mean difference ± SD	ICC	p-value	Mean difference ± SD	ICC	*p-value*
2D global RV-Sl	1.8±7.5	0.88	0.007	2.0±8.9	0.82	0.02
2D apical RV-Sl	1.5±1.1	0.96	0.001	2.8±3.1	0.92	0.01
2D mid RV-Sl	2.0±1.2	0.85	0.01	3.0±2.1	0.79	0.03
2D basal RV-Sl	1.9±1.5	0.87	0.009	2.5±1.2	0.80	0.03

2D RV-Sl, two dimensional right ventricular longitudinal strain; ICC, intraclass correlation coefficient; SD, standard deviation.

## Discussion

The main findings of our study are as follows:

- OSA has a detrimental effect on LV and RV function, which increases with severity of disease. (ii) This effect is in part reversible under effective OSA therapy and (iii) patients with severe OSA and associated myocardial dysfunction benefit most from CPAP therapy.

To the best of our knowledge, this is one of the first studies to examine the impact of severe OSA on global and regional right ventricular function as determined with 2D ST and the effect of CPAP therapy on measurable right ventricular functional parameters.

### Severity of OSA and LV/RV Dysfunction

The impact of obstructive sleep apnea on left and right ventricular function and structure is controversial. Theoretically, OSA relevantly influences RV and LV hemodynamics by an increase of pulmonary artery pressure during apnea episodes [17], [18]. Severe hypoxemia, hypercapnia and acidosis lead to sympathetic activation and therefore to sympathetic mediated vasoconstriction during apnea episodes increasing systolic blood pressure, vascular shear stress and consequently the risk for myocardial infarction [19], [20]. However, evidence from previous studies assessing right ventricular function in OSA patients is inconsistent with a wide range of OSA dependent right ventricular alterations which were detected in 0–70% of patients included [21]–[23].

In the Framingham Heart study, the effect of sleep-disordered breathing on right heart structure and function was examined by Guidry and colleagues. They demonstrated that diameters of the RV cavities of the right heart and LV systolic function were not significantly diverging between two groups of patients with different severity of sleep disordered breathing [24]. On the other hand, Romero-Coral and colleague showed that OSA leads to impaired right and left ventricular function determined with MPI in 85 OSA patients undergoing their first overnight polysomnography [25]. Sanner and colleagues reported a significant correlation between AHI and right ventricular ejection fraction measured with radionuclide ventriculography in 107 patients [26]. Recently Colish and colleagues found right ventricular and atrial diameters positively influenced by 12 months of CPAP therapy in a cohort of 47 OSA patients [27].

The impact of OSA on systolic and diastolic LV function has been largely addressed by several authors [28], [29]. The positive effect of CPAP therapy on left ventricular function was shown by Shivalkar et al. They proved that interventricular septum thickness, left ventricular stroke volume and right ventricular tissue Doppler systolic velocity increase after effective CPAP therapy [30]. Butt and colleagues were able to prove a positive effect of CPAP on LV function using 2-dimensional echocardiography, tissue Doppler imaging, and 3-dimensional echocardiography in subjects with moderate-severe OSA, compared to a matched patient cohort without OSA [31].

This complies with our findings of structural and functional cardiac alterations in patients with sleep related breathing disorder and the favorable effects of CPAP.

The results of a recently published study by Altekin and colleagues are, also, in line with our findings, that there is a strong correlation of RV strain and the severity of OSA. In this study RV strain was even able to detect RV dysfunction in a subclinical phase of RV function deterioration [32].

The partially discrepant findings from the cited studies concerning RV functional parameters might be due to the fact that follow up time as well as definition of OSA were not consistently defined. Furthermore, the determination of RV function is not standardized. Indirect parameters for deteriorated RV function such as RV diameter or wall diameters might not be eligible to monitor early and subtler changes in RV function.

BMI was significantly related to AHI frequency and was the only demographic parameter shown to be independently associated with the severity of OSA. Nevertheless, the correlation between apical 2D RV-Sl and AHI frequency was stronger as confirmed with comparative ROC curve analysis. This emphasizes the independent and important relation of apical RV impairment and severity of OSA.

### 2D Strain and RV Function

2D strain imaging has been established for assessment of regional left and right ventricular function and offers a good correlation with right ventricular ejection fraction as well [33], [34]. It is known to be of excellent value to evaluate LV regional and global function. 2D ST has a good inter- and intra-observer agreement, is well correlated to MRI and highly reproducible [35]. Speckle-tracking analysis derived 2 dimensional strain values are independent from the angle of insonation [36], and therefore offer reasonable advantages over TDI for the determination of regional wall abnormalities [37], [38].

Furthermore, 2D strain derived parameters have been found superior to conventional echocardiographic parameters for identification of patients with severe pathological alterations in RV function such as arrhythmogenic right ventricular dysplasia (ARVD) [39] and for the monitoring of medical treatment of severe pulmonary hypertension (PH) [40].

In our study on patients with different stages of OSA, we found a significant correlation of regional and global RV strain parameters with the severity of OSA and of note, a relevant increase of RV deformation capabilities after CPAP therapy. Our data indicates that apical RV function alteration might be a sensitive parameter to detect early and even subclinical deterioration in RV function.

These findings are in line with the results from Fernandez-Friera et al. who used CMR for RV function imaging in a cohort of 192 patients with different stages of pulmonary hypertension (PH). The author and colleagues found that basal RV ejection fraction (RVEF) did not differ between patients with mild or moderate PH. However, they showed that patients with moderate PH had significantly lower apical RVEF, with an apical RVEF<50% that was more sensitive than global RV dysfunction for the detection of PH [41]. In a study by López-Candalez et al. using TDI and TDI derived strain in patients with severe PH, they found apical longitudinal strain most predictable for adverse outcome in those patients [42].

### Clinical Impact

Since OSA is closely related to left and right ventricular dysfunction which improves with effective CPAP therapy, we suggest an early start of CPAP therapy in all patients with relevant OSA to prevent permanent impairment of the left and right ventricular function and structure.

### Limitations

The study is limited by its not randomized, not blinded and single side character. Our sleep clinic cohort may not reflect the findings in the general community because of the limited number of patients. Our sleep clinic cohort may not reflect the findings in the general community because of the limited number of patients. Whilst significant cardiovascular co morbidities were present at baseline in our study cohort we can not fully exclude that changes in RV or LV function could have been mediated by an indirect effect of CPAP therapy on these co morbidities. The observational design of the study does not allow for this concern to be addressed. Furthermore, there are many other confounding factors that might alter global and regional RV function in the general population. Our conclusion applies therefore specifically to a pre-selected population with proven OSA.

### Conclusion

OSA seems to have deteriorating impact on left and right ventricular function. After 6 months of therapy, we found a beneficial effect of CPAP on LV and RV functional parameters which was pronounced in patients with severe OSA. 2D speckle tracking might be of value to determine early changes in global and regional right ventricular function.

## Supporting Information

Table S1 **Multivariate stepwise regression analysis of all echocardiographic parameters and baseline demographics.**
(DOCX)
