# Supplementary material for: Impact of Untreated Obstructive Sleep Apnea on Left and Right Ventricular Myocardial Function and Effects of CPAP Therapy
Source: PLoS One. 2013 Oct 11;8(10):e76352. doi: 10.1371/journal.pone.0076352 (PMC3795765; doi:10.1371/journal.pone.0076352)
Supplement: Table S1 — Multivariate stepwise regression analysis of all echocardiographic parameters and baseline demographics. (DOCX) [file pone.0076352.s001.docx]

**Supporting information**

***Table S1***

Multivariate stepwise regression analysis of all echocardiographic parameters and baseline demographics

| **Variable** | **Univariate regression** | | | | **Multivariate regression** | | |
| --- | --- | --- | --- | --- | --- | --- | --- |
|  | **B** | **OR** | **95% CI** | **P** | **HR** | **95% CI** | **P** |
| Age | 0.125 | 1.1 | 0.6 – 1.1 | 0.9 |  |  |  |
| Male gender | 0.018 | 1.1 | 0.9 – 1.2 | 0.4 |  |  |  |
| BMI | 0.806 | 4.2 | 1.0 – 17.8 | 0.01 | 3.2 | 1.5 – 6.7 | 0.0007 |
| ODI | -1.005 | 0.3 | 0.03 – 2.6 | 0.4 |  |  |  |
| ESS | 0.473 | 0.6 | 0.2 – 1.9 | 0.5 |  |  |  |
| Hypertension | 0.304 | 1.5 | 0.5 – 2.3 | 0.5 |  |  |  |
| CHF | 0.282 | 1.1 | 0.4 – 5.6 | 0.6 |  |  |  |
| Diabetes mellitus | -1.201 | 0.45 | 0.07 – 3.1 | 0.5 |  |  |  |
| History of stroke | 0.176 | 0.7 | 0.4 – 1.7 | 0.9 |  |  |  |
| CAD | 0.402 | 1.3 | 0.7 – 2.4 | 0.8 |  |  |  |
| Smoking | 0.423 | 1.1 | 0.12 – 3.4 | 0.7 |  |  |  |
| HLP | 0.531 | 2.2 | 0.4 – 4.1 | 0.6 |  |  |  |
| LVEF | 0.211 | 1.0 | 0.3 – 0.9 | 0.9 |  |  |  |
| sPAP | 0.023 | 1.4 | 0.05 – 2.0 | 0.5 |  |  |  |
| IVSd | 0.278 | 1.7 | 0.4 – 4.2 | 0.7 |  |  |  |
| SV | -1.212 | 1.4 | 0.07 – 3.1 | 0.6 |  |  |  |
| MV e´/a´ | 0.198 | 1.0 | 0.02 – 1.9 | 0.6 |  |  |  |
| LV MPI | 0.089 | 1.5 | 0.8 – 2.7 | 0.7 |  |  |  |
| e/e´ | 0.359 | 0.9 | 0.7 – 3.3 | 0.8 |  |  |  |
| RV MPI | -1.003 | 1.1 | 0.09 – 1.8 | 0.6 |  |  |  |
| TDI TKS | 0.396 | 1.2 | 0.9 – 1.9 | 0.7 |  |  |  |
| TAPSE | 0.201 | 1.3 | 1 – 2.9 | 0.6 |  |  |  |
| 2D global RV-Sl | 0.632 | 1.4 | 0.7 – 2.6 | 0.6 |  |  |  |
| 2D apical RV-Sl | 3.785 | 5.1 | 3.1 – 8.4 | 0.001 | 4.5 | 2.9 – 7.1 | <0.0001 |
| 2D medial RV-Sl | 0.211 | 1.4 | 0.8 – 2.4 | 0.5 |  |  |  |
| 2D basal RV-Sl | 0.091 | 1.6 | 0.6 – 4.2 | 0.8 |  |  |  |

*2D RV-Sl, two dimensional right ventricular longitudinal strain; ACEI, angiotensin converting enzyme inhibitors; AHI, apnea hypopnea index; ARBI, angiotensin receptor blocker; CAD, coronary artery disease; CHF, chronic heart failure; BMI, body mass index; e/e´, early/early´; ESS, Epworth sleepiness scale; HLP, hyperlipoproteinemia; IVSd, diastolic interventricular septum thickness; LVEF, left ventricular ejection fraction; LV/RV MPI, left ventricular/right ventricular myocardial performance index; MV e´/a, mitral velocity early´/atrial; ns, not statistically significant; ODI, oxygen desaturation index; SD, standard deviation; sPAP, systolic pulmonary artery pressure; SV, stroke volume; TAPSE, tricuspid annular plane systolic excursion; TDI TKS, tricuspid annular systolic tissue Doppler velocity*
